# What drives consumer participation in virtual CSR? The impact of external scenarios

**DOI:** 10.1371/journal.pone.0342470

**Published:** 2026-02-11

**Authors:** Fan Yang, Yuting Song, Jinyi Hu, Huiying Zhang

**Affiliations:** 1 School of Management Science and Engineering, Nanjing University of Finance and Economics, Nanjing, China; 2 School of Mathematical Sciences, East China Normal University, Shanghai, China; 3 China Overseas (Hong Kong) Limited, Hong Kong, China; 4 Faculty of Construction and Environment, The Hong Kong Polytechnic University, Hong Kong, China; 5 College of Management and Economics, Tianjin University, Tianjin, China; Università degli Studi di Milano: Universita degli Studi di Milano, ITALY

## Abstract

The proliferation of social media has transformed the fulfillment of corporate social responsibility (CSR) from traditional offline activities to emerging online methods, ushering in the era of Virtual CSR activities. This study focuses on the crucial issue of enhancing consumer engagement in Virtual CSR initiatives. Drawing upon the Theory of Planned Behavior and Perceived Risk Theory, this study empirically analyzes the factors influencing consumers’ willingness and behavior to participate in Virtual CSR activities through questionnaire surveys. Our findings reveal that behavioral attitudes, perceived behavioral control, and external scenarios positively impact consumer willingness to participate, which in turn significantly promotes participation behavior. Furthermore, willingness to participate serves as a mediator between these factors and actual participation behavior. Based on these insights, we propose practical recommendations for enterprises to optimize their Virtual CSR strategies.

## 1. Introduction

The rapid evolution of technology has profoundly altered the landscape of social media, making it an ubiquitous and essential aspect of contemporary life. Consumers engage with companies through social media platforms, thereby transforming the way in which companies fulfill their social responsibility. The emergence of virtual corporate social responsibility (virtual CSR) represents a paradigm shift from offline to online methods. Virtual CSR activities involve enterprises leveraging the Internet and social media to engage stakeholders in CSR endeavors [1,2]. While not inherently green economy initiatives, they are largely highly associated with the green economy and can serve as significant drivers for its development. The green economy is not merely an academic concept, but a practical pathway towards planetary resilience [3]. In the current digital era, several virtual corporate social responsibility (CSR) activities have gained significant popularity. Notably, these include the “Ant Forest” and “Ant Manor” initiatives launched on the Alipay platform, the “Donate Steps” activity on the WeChat platform, the “Oasis Forest” project on the Weibo platform, and PepsiCo’s “Refresh Everything” campaign. These activities represent innovative approaches to engaging the public in social responsibility efforts through digital means.

In virtual CSR, social media acts as a bridge between consumers and enterprises, eliminating the traditional offline information transmission and feedback loops [4]. This direct participation fosters trust in enterprises and facilitates the resolution of issues related to social value creation. For instance, Ant Group’s 2023 Sustainability Report highlights the success of initiatives like “Alipay Baba Farm” and “Ant Forest” in engaging millions of users in eco-friendly practices. The Ant Forest project has successfully ingrained the concept of a green and low-carbon lifestyle into people’s minds, generating a significant amount of “green energy”, in which enterprises contributed by funding tree planting efforts, with users planting a large number of trees, covering an extensive area. In addition to the Ant Forest project, the WeChat Donate Steps and other virtual CSR activities have achieved notable success, not only enabling companies to successfully fulfil their social responsibility but also fulfilling consumers’ desire to be socially responsible.

As an increasing number of companies achieve their goals through virtual CSR campaigns, the advantages of such campaigns are becoming more widely recognized. The ease of understanding and participating in various activities through social media, or what might be termed usability, can reduce cognitive load and enhance user satisfaction, ultimately leading to higher engagement [5]. Nevertheless, not all virtual CSR activities are capable of achieving the overarching objective of the enterprise. The pivotal factor determining the success of virtual CSR activities is the extent to which consumers actively participate. Consequently, enterprises must prioritize strategies to enhance consumer initiative and motivation to participate in virtual CSR activities.

Traditionally, enterprises are the main entities to fulfill social responsibility, and the main way is ordinary fundraising. Previous studies mainly focus on the participation of enterprises, while paying relatively little attention to the participation of consumers and the role they play in the process of fulfilling social responsibility, as well as consumers’ behavior [6,7]. Meanwhile, virtual CSR activities are a newly emerging way of CSR fulfillment in recent years due to the increasing activitise of social media, and the number of studies related to virtual CSR activities is relatively small. Most studies on the factors affecting consumer willingness to participate in virtual CSR activities approach the research from aspects such as the gamification of virtual CSR activities [8], interactive paths [4], and consumers’ perceived value. Little research has been conducted on consumer willingness and behavior in participating in virtual CSR activities from the perspectives of the Theory of Planned Behavior and the Perceived Risk Theory [9,10]. Therefore, this study positions consumers as the central players in virtual CSR activities and investigates the impact of various factors on consumer willingness and behavior in participating in virtual CSR activities based on these two theories.

The theory of planned behaviour (TPB) serves as a core framework for explaining individual behavioral decision-making, enjoying extensive application. By introducing new variables, its predictive power regarding behavioral intentions can be further enhanced [11]. Its core logic emphasizes the driving role of cognitive factors in shaping behavioral intentions, reflecting rational individuals’ tendency to maximize behavioral benefits. However, in uncertain digital philanthropic activities such as virtual CSR participation, customer decisions are influenced not only by positive motivations but also modulated by risk perception. Perceived risk theory (PRT) aptly addresses the loss-aversion perspective. Integrating both theories forms a dual-channel motivation-risk decision model, better aligning with the practical decision-making logic observed in behavioral economics. Current attempts to integrate these theories often treat risk merely as an external variable of TPB, failing to establish a bidirectionally interactive theoretical framework [12]. Combining TPB with PRT is not a simple variable superposition but rather an integrated analytical framework built upon the intrinsic complementarity of these two theories’ explanatory dimensions, offering greater interpretative tension and predictive precision. This theoretical fusion not only addresses the practical demands of increasingly complex behavioral decision-making in the digital age but also provides a scientific diagnostic tool for resolving the practical dilemma of high attention yet low conversion rates in virtual CSR.

**Fig 1:**
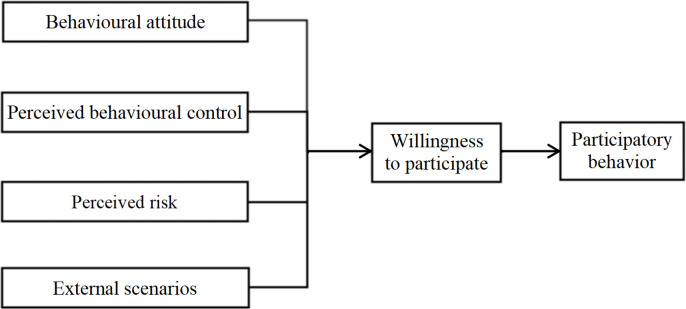
Conceptual model.

In comparison to the conventional approach, virtual CSR activities are characterized by greater interactivity. They are able to attract consumers who are keen to participate in order to obtain relevant information, lessen misunderstanding in the process of information transmission, and facilitate the achievement of desired outcomes for enterprises. However, due to the absence of face-to-face interaction, there is a certain degree of anonymity, and consumers are at liberty to choose whether to participate or not. Consequently, companies engaged in virtual CSR activities are confronted with a shared challenge: how can they effectively attract consumers to participate in these activities? This study employs empirical data to examine the factors influencing consumer participation in virtual CSR activities, with the aim of providing recommendations to companies on the design of such activities, with the ultimate goal of enhancing consumer willingness to engage with these activities. This study is based on the Theory of Planned Behavior (TPB) model [13,14] and introduces the Perceived Risk Theory [15] to construct the model, which consists of six variables: behavioral attitude, perceived behavioral control, perceived risk, external scenarios, willingness to participate, and participatory behavior. The requisite data are collected through questionnaire surveys and collection, and the influence of each factor on consumer participation in virtual CSR activities is obtained through the collation and analysis of the data. In light of the findings yielded from the empirical investigation, the study provides countermeasures and suggestions for enterprises engaged in virtual CSR activities. The conceptual model of this study is presented in Fig 1.

## 2. Literature review and hypotheses development

### 2.1. Behavioural attitude, perceived behavioural control, external scenarios and willingness to participate

Morsing et al. (2016) posited that stakeholders play an instrumental role in CSR activities [16]. Attracting stakeholders to participate in CSR activities and assume a communication role can further enable companies to gain a favorable reputation. Moreover, consumers are identified as the most influential type of stakeholder. Over time, scholars have observed that the role of stakeholders in CSR activities has become increasingly prominent, leading to the proposal of the concept of CSR co-creation [4]. In the context of CSR co-creation, the primary focus is no longer confined to the enterprise alone. Instead, it encompasses the enterprise in collaboration with its stakeholders, with the objective of creating value for the enterprise, society, and other stakeholders. The success of CSR activities is contingent upon this value creation process, which is a pivotal aspect of social value creation [17].

The advent and proliferation of social media have diminished the communication barriers between companies and consumers, concomitantly giving rise to the phenomenon of virtual corporate social responsibility (CSR) activities. Korschun & Du (2013) define a virtual CSR activity as a process in which a company deliberately leverages social media platforms to engage stakeholders in actively contributing to the company’s social responsibility program, wherein stakeholders and the company collectively generate multifaceted value [1]. Furthermore, they posit that the most pivotal element is to motivate stakeholders to engage actively in corporate CSR activities [1]. Peloza & Shang (2011) elucidated the distinction between virtual CSR activities and traditional CSR activities through an examination of four key aspects: user role, user participation mode, user resources and activity process [18]. In this context, the user is positioned as a principal actor in the activity, rather than merely a financial contributor. Instead, the user is emotionally invested in the activity through the use of gamification, and the process of the virtual CSR activity is rendered more transparent [18]. Additionally, Kim (2017) examined the data and indicated that the function of communication in virtual CSR activities has a beneficial impact on consumer awareness of CSR, confidence in the company, and the perception of a favorable image of the company [19].

The theory of planned behavior (TPB) provides an explanation and prediction of human behavior from a social psychological perspective. It posits that an individual’s willingness to engage in positive and proactive behavior plays a pivotal role in the generation of behavior. Furthermore, it postulates that willingness to behave serves as an intermediate bridge between all the factors that have the potential to influence an individual’s behavior and the behavior itself. In TPB, the factors influencing behavioral intentions include behavioral attitudes, subjective norms and perceived behavioral control. The TPB model has also been widely applied in researching consumer willingness and behavior in participating in Internet – related public welfare activities, which serves to demonstrate the scientific and reasonable nature of the model. Steg & Vlek (2009) found that the public’s willingness to participate in “Internet + Tree Planting” under the background of carbon neutrality will be influenced by behavioral attitudes and perceived behavioral control [20]. Thøgersen (2007) found that perceived behavioral control and external scenarios positively affect college students’ willingness to participate in the recycling of used mobile phones [21]. Cialdini & Goldstein (2004) pointed out that the more positive the consumer’s attitude, the stronger the subjective norms and perceived behavioral control, the stronger the consumer’s willingness to share information about micro-community welfare crowdfunding in the circle of friends [22]. All of the above studies have used the TPB model, which proves that the three assumptions embedded in the TPB model are also true for the willingness and behavior of participating in Internet – related public welfare activities.

Virtual CSR activities also constitute one of the forms of Internet public welfare activities. Consumer willingness to participate in virtual CSR activities and their behaviors are also quite similar to those in other Internet public welfare activities. This is evidenced by the fact that they are also affected by behavioral attitudes, perceived behavioral control and subjective norms. The term “subjective norms” primarily denotes the impact of others’ decisions and awareness on an individual’s own choices. In this study, the concept of subjective norms is simplified to encompass external situational factors. In light of the above, this study puts forth the following hypotheses:

H1: Behavioral attitude positively influences consumer willingness to participate in virtual CSR.

H2: Perceived behavioral control positively influences consumer willingness to participate in virtual CSR.

H3: External scenarios positively influence consumer willingness to participate in virtual CSR.

### 2.2. Perceived risk and consumer willingness to participate

The concept of perceived risk was first introduced in the field of psychology and subsequently applied to the marketing field [15]. This theory posits that it is challenging to accurately predict the final outcome of a consumer’s decision – making process due to the multitude of influencing factors involved. Some of these factors may ultimately lead to consumer dissatisfaction [23]. Stone & Gronhaug (1993) argued that the types of perceived risk encountered by consumers when engaging in online consumption can be broadly classified into three categories: service quality risk, personal information risk, and product quality risk [24]. The classification of perceived risk is not unique, as scholars may categorize it differently depending on the context. Mitchell (1999) proposed a classification of perceived risks associated with the purchase of goods, including product quality risk, product safety risk, and economic benefit risk [25]. Cheung & Lee (2005) determined that the factor influencing the consumer’s inclination to accept network algorithm services, as opposed to their behavior, is the perceived risk by employing a conditional algorithm [26]. In other words, an elevated perceived risk results in a diminished user willingness to accept network algorithm recommendation services. Yin et al. (2019) took the emerging social commerce as the research background, confirmed that the consumer’s trust in the seller and the perceived risk will have a significant impact on their subsequent purchase willingness, which is consistent with the findings in traditional commerce [27]. Wilson (2000) found, based on meta-analysis, that perceived risk would affect users’ online information search willingness [28]. Acquisti et al. (2015) pointed out that perceived risk would directly affect the behavior of mobile APP users’ private information settings [29]. Therefore, perceived risk arises when consumers are unsure of which behavior to choose to achieve the most satisfactory outcome. Consumers often want to achieve their desired goals, but the presence of perceived risk creates uncertainty about the outcome. Therefore, the following hypothesis is proposed in this study:

H4: Perceived risk negatively influences consumer willingness to participate in virtual CSR.

### 2.3. Consumer willingness to participate and participatory behavior

The conduct of virtual CSR activities does not directly cause consumers to participate, as a person facing a choice of whether to take action is primarily influenced by their subjective will. The most effective tool for predicting behavioural decision – making is behavioural willingness [30]. In order for consumers to participate in virtual CSR activities, they must first increase their awareness and positive perception of such activities. This is followed by the gradual development of subjective preference for virtual CSR activities, which then leads to a willingness to participate. As this willingness to participate increases, the likelihood of consumers engaging in virtual CSR activities also increases. It can therefore be posited that the more positive consumers’ attitudes towards virtual CSR activities are, the greater the likelihood that their willingness to participate will be transformed into actual participation. The role of hedonistic motivation and perceived enjoyment in influencing green consumption behaviour indicates that individuals derive pleasure and satisfaction from participating in activities that promote sustainable development [31]. Furthermore, numerous previous studies in disparate fields have also demonstrated that behavioral intention is a significant predictor of behavior [32]. In light of the above, the following hypotheses are put forth in this study:

H5: Consumer willingness to participate positively influences participatory behavior in virtual CSR.

H6: Consumer willingness to participate mediates the relationship between behavioural attitudes and participatory behavior in virtual CSR.

H7: Consumer willingness to participate mediates the relationship between perceived behavioural control and participatory behavior in virtual CSR.

H8: Consumer willingness to participate mediates the relationship between perceived risk and participatory behavior in virtual CSR.

H9: Consumer willingness to participate mediates the relationship between external scenarios and participatory behavior in virtual CSR.

## 3. Research methodology

### 3.1. Questionnaire design

A questionnaire was constructed based on the existing literature from mature scales and the variables in the theoretical model established in this study. The questionnaire was designed to assess consumer willingness and behaviours regarding participation in virtual CSR activities in the context of “Internet + Public Welfare”. The primary focus of the questionnaire is the measurement of each variable within the theoretical model, which encompasses six variables: behavioural attitude, perceived behavioural control, perceived risk, external scenarios, willingness to participate and participatory behavior. This is presented in a total of 19 questions. The scale is scored using a five-point Likert scale, in which the degree of agreement of the questionnaire participants with regard to the items is divided into five categories: strongly disagree, relatively disagree, uncertain, relatively agree, and strongly agree, with scores of 1–5 assigned to each category respectively. Table 1 presents measurement items.

**Table 1:** Measurement items.

Construct	Measurement items	References
Behavioral attitudes (TD)	TD1. I am positive about participating in virtual CSR activities.	[20,21,33]
	TD2. I think participation in virtual CSR activities has positive benefits.	
	TD3. I think participating in virtual CSR activities can bring benefits.	
Perceived behavioral control (ZJ)	ZJ1. I think participating in virtual CSR activities is an easy and simple thing to do.	[21,33]
	ZJ2. I have enough time to participate in virtual CSR activities.	
	ZJ3. I have easy access to information about the virtual CSR activities.	
Perceived risk (FX)	FX1. I am concerned that I will not be able to achieve the expected results after participating in the virtual CSR program.	[20,29]
	FX2. I am concerned that participating in the virtual CSR program will require too much time and effort.	
	FX3. I am concerned about the risk of disclosing my personal information when participating in the virtual CSR programme.	
External scenario (QJ)	QJ1. People around me would motivate me to participate in virtual CSR activities.	[33]
	QJ2. I would be motivated to participate in virtual CSR activities by media reports.	
	QJ3. I would be motivated to participate in virtual CSR activities by expert opinions.	
Willingness to participate (YY)	YY1. I am willing to participate in virtual CSR activities actively now.	[20,33]
	YY2. I would like to be actively involved in virtual CSR activities in the future.	
	YY3. I am prepared to continue to follow the development of virtual CSR activities.	
	YY4. I am willing to recommend virtual CSR activities on the platform to people around me.	
Participatory behavior (YW)	XW1. I have participated in virtual CSR activities in the past.	[33]
	XW2. I am continuing to engage in virtual CSR activities.	
	XW3. I have recommended virtual CSR activities to people around me.	

All participants provided informed consent prior to participating in the study. The consent was obtained in an online explicit form: participants voluntarily clicked “Start” to fill out the questionnaire after reading the informed consent statement, which was documented through the system log of the questionnaire platform. The study was conducted according to the guidelines of the Declaration of Helsinki and approved by the Institutional Review Board of School of Management Science and Engineering, Nanjing University of Finance and Economics (protocol code [2022]-73 and 17 November 2022). All participants provided informed consent prior to participating in the study. The consent was obtained in an online explicit form: participants voluntarily clicked “Start” to fill out the questionnaire after reading the informed consent statement, which was documented through the system log of the questionnaire platform. No minors were involved in this study, so parental or guardian consent was not required.

### 3.2. Data collection

The questionnaire distribution channels of this study were the Internet platforms WeChat, QQ, Weibo, PostBar, Xiaohongshu and other social software, which yielded 232 valid questionnaires. The recruitment period for this study lasted from 15 March 2023–30 June 2023. As illustrated in Table 2, the proportion of males in the sample data was 41.4%, while the proportion of females was 58.6%. Additionally, the majority of respondents were young adults between the ages of 20 and 25, comprising 64.7% of the total sample. The remaining 35.3% were distributed among individuals under 20 years old (13.4%) and those aged 26 years and above (21.6%). The majority of respondents held a Bachelor’s degree, and the majority reported an income of 3,000 yuan or less. In light of the aforementioned data, it can be surmised that the majority of respondents are college students. Consequently, the data collected from this questionnaire is more reflective of the reality that the primary users of social media are young people.

**Table 2:** Statistical analysis of basic information.

Variable	Option	Frequency	Percentage
Sex	Male	96	41.4
	Female	136	58.6
Age	Below 20 years old	31	13.4
	20-25 years old	150	64.7
	26-30 years old	27	11.6
	30-35 years old	10	4.3
	Above 35 years old	14	6.0
Academic qualification	High school and below	22	9.5
	College degree	13	5.6
	Undergraduate degree	174	75.0
	Postgraduate and above	23	9.9
Average monthly income	3000 and below	151	65.1
	3000-6000	42	18.1
	6000-10000	22	9.5
	10000 or above	17	7.3

### 3.3. Reliability and validity tests

The results of the reliability analyses are presented in Table 3. The majority of the reliability coefficients for the dimensions in the scale fall within the range of 0.8−1, with only one item falling within the range of 0.7–0.8. It can therefore be concluded that the scale used in this study exhibits good internal consistency and reliability. A CITC value below the standard value of 0.3 indicates an inappropriateness in the measurement question item, necessitating its deletion. As evidenced in Table 3, the CITC values of all measurement items exceed 0.3, and the Cronbach’s α of each item when the item is deleted are less than the overall Cronbach’s α of their corresponding dimensions. This indicates that the measurement item settings are reasonable and can be utilized for subsequent analyses.

**Table 3:** Results of questionnaire scale reliability test.

Construct	Item code	CITC	The α factor for which the item has been removed	Cronbach’ α
Behavioral attitudes	TD1	0.770	0.830	0.881
	TD2	0.783	0.819	
	TD3	0.755	0.843	
Perceived behavioral control	ZJ1	0.653	0.742	0.809
	ZJ2	0.674	0.720	
	ZJ3	0.644	0.751	
Perceived risk	FX1	0.702	0.727	0.821
	FX2	0.706	0.720	
	FX3	0.619	0.809	
Exterior scenarios	QJ1	0.651	0.821	0.836
	QJ2	0.701	0.770	
	QJ3	0.760	0.712	
Willingness to participate	YY1	0.763	0.828	0.875
	YY2	0.706	0.850	
	YY3	0.744	0.837	
	YY4	0.721	0.845	
Participatory behavior	XW1	0.574	0.743	0.776
	XW2	0.640	0.668	
	XW3	0.636	0.671	

The measurement items in the scale used in this study were derived from mature scales at home and abroad, which were subsequently modified to align with the actual research content. The resulting questionnaire scale was deemed to possess satisfactory content validity. The structural validity test is primarily composed of two distinct components: the examination of the internal relationships within the questionnaire scale and the assessment of the correspondence between the measurement items and the variables to which they pertain. Prior to conducting factor analysis, it is necessary to perform the KMO test and Bartlett’s test on the data. In general, the validity level is deemed to be satisfactory when the KMO sampling adequacy value is greater than 0.7. Additionally, if the relationship between variables is significantly correlated, the p-value of the Bartlett’s test of sphericity will be less than 0.05. As illustrated in Table 4, the KMO value of the data from the present study is 0.879, which exceeds the standard value of 0.7. Additionally, the p-value of the Bartlett’s test of sphericity is less than 0.05, indicating that the data collected in the study exhibit a significant correlation and are thus suitable for factor analysis.

**Table 4:** Results of KMO and Bartlett’s test.

KMO		0.879
Bartlett’ sphericity test	Approximate chi-square	2461.289
	df	171
	p	0.000

Six factors were forcedly extracted from the 19 items of this scale according to the number of fixed factors. The results of the analysis are presented in Table 5. The total variance explained by these six factors for the 19 items is 75.683%, which exceeds the standard value of 60%. Therefore, it can be assumed that the six factors designed for this scale are able to interpret the content of the original data to a satisfactory extent.

**Table 5:** Explanation of total variance.

Ingredients	Initial eigenvalues			Sum of squared loadings extracted			Sum of squared loadings rotated		
	Total	Variance percentage	Cumulative %	Total	Variance percentage	Cumulative %	Total	Variance percentage	Cumulative %
1	7.510	39.527	39.527	7.510	39.527	39.527	2.985	15.709	15.709
2	2.205	11.607	51.133	2.205	11.607	51.133	2.517	13.246	28.955
3	1.541	8.111	59.245	1.541	8.111	59.245	2.496	13.137	42.093
4	1.256	6.609	65.854	1.256	6.609	65.854	2.265	11.923	54.016
5	1.119	5.889	71.743	1.119	5.889	71.743	2.173	11.437	65.453
6	0.748	3.939	75.683	0.748	3.939	75.683	1.944	10.230	75.683
7	0.568	2.987	78.670						
8	0.548	2.882	81.552						
9	0.503	2.645	84.197						
10	0.456	2.399	86.596						
11	0.428	2.253	88.849						
12	0.349	1.838	90.687						
13	0.326	1.714	92.401						
14	0.307	1.616	94.017						
15	0.267	1.404	95.421						
16	0.246	1.297	96.718						
17	0.218	1.149	97.867						
18	0.215	1.133	99.001						
19	0.190	0.999	100.000						

Extraction method: Principal component analysis.

In the exploratory factor analysis of this study, the results of the rotated component matrix were set to show only coefficient values greater than 0.5, and the results are presented in Table 6. All the question items in Table 6 had factor loadings greater than the specified criterion of 0.5 on their corresponding common factor dimensions, indicating that the items under the same dimension are more closely related to their corresponding factors and that the structural validity of this scale is good.

**Table 6:** Rotated component matrix.

Item	Ingredients					
	1	2	3	4	5	6
YY1	0.781					
YY3	0.772					
YY4	0.759					
YY2	0.720					
TD2		0.842				
TD3		0.809				
QJ2			0.817			
QJ3			0.796			
QJ1			0.682			
FX1				0.852		
FX3				0.844		
FX2				0.837		
ZJ1					0.813	
ZJ2					0.800	
ZJ3					0.644	
XW1						0.796
XW2						0.712
XW3						0.587

As illustrated in Table 7, the majority of scale items exhibit factor loading coefficients that exceed the established threshold of 0.7. However, the factor loadings of FX3 and XW1 are 0.679 and 0.646, respectively, yet remain in close proximity to the established threshold. Removing them would narrow the conceptual domain being measured and potentially introduce construct deficiency. Meanwhile, the AVE values of all dimensions exceed the standard value of 0.5, and the CR value is above the standard value of 0.7. Therefore, it can be concluded that the scale employed in the present study exhibits satisfactory convergent validity.

**Table 7:** Convergence validity and combined reliability test of each dimension of the scale.

Path relationship			Estimate	AVE	CR
TD3	←	Behavioral attitudes	0.833	0.712	0.881
TD2	←	Behavioral attitudes	0.858		
TD1	←	Behavioral attitudes	0.840		
ZJ3	←	Perceived behavioral control	0.793	0.583	0.807
ZJ2	←	Perceived behavioral control	0.769		
ZJ1	←	Perceived behavioral control	0.727		
FX3	←	Perceived risk	0.679	0.611	0.824
FX2	←	Perceived risk	0.843		
FX1	←	Perceived risk	0.813		
QJ3	←	Exterior scenarios	0.869	0.641	0.842
QJ2	←	Exterior scenarios	0.775		
QJ1	←	Exterior scenarios	0.753		
YY4	←	Willingness to participate	0.792	0.640	0.877
YY3	←	Willingness to participate	0.798		
YY2	←	Willingness to participate	0.775		
YY1	←	Willingness to participate	0.834		
XW3	←	Participatory behavior	0.801	0.541	0.778
XW2	←	Participatory behavior	0.750		
XW1	←	Participatory behavior	0.646		

As shown in Table 8, the Pearson correlation coefficients between each pair of dimensions in this test of discriminant validity are less than the square root value of the AVE corresponding to that dimension. This indicates that there is a satisfactory discriminant validity between each pair of dimensions.

**Table 8:** Pearson correlation coefficient and AVE square root value.

Construct	Behavioral attitudes	Perceived behavioral control	Perceived risk	Exterior scenarios	Willingness to participate	Participatory behavior
Behavioral attitudes	**0.844**					
Perceived behavioral control	0.503	**0.764**				
Perceived risk	0.225	0.208	**0.782**			
Exterior scenarios	0.440	0.542	0.276	**0.801**		
Willingness to participate	0.476	0.502	0.190	0.593	**0.800**	
Participatory behavior	0.409	0.513	0.049	0.480	0.670	**0.735**

Note: The bold digits in the diagonal are the square root values of the AVE.

## 4. Data analysis and results

### 4.1. Normality test

The skewness and kurtosis of the question items of each scale were measured to ascertain whether the data conformed to an approximate normal distribution. In accordance with the normality test criteria proposed by Kline et al. (1998, 2011), the data is deemed to conform to an approximate normal distribution if the absolute value of the skewness coefficient is less than 3 and the absolute value of the kurtosis coefficient is less than 8 [34,35]. The results of the analyses, as presented in Table 9, indicate that the absolute values of the skewness and kurtosis coefficients for each of the measurement question items in this study are within the specified ranges. Consequently, the data for each of the measurement question items can be considered to conform to an approximate normal distribution and can be used to construct the structural equation model and conduct hypothesis testing.

**Table 9:** Results of normality test.

Construct	Item code	Skewness	Kurtosis
Behavioral attitudes	TD1	−1.159	2.301
	TD2	−1.139	2.214
	TD3	−0.886	1.654
Perceived behavioral control	ZJ1	−0.794	0.734
	ZJ2	−0.775	0.574
	ZJ3	−0.693	0.641
Perceived risk	FX1	−0.531	−0.462
	FX2	−0.300	−0.736
	FX3	−0.388	−0.445
Exterior scenarios	QJ1	−1.084	1.440
	QJ2	−0.435	−0.242
	QJ3	−0.594	−0.359
Willingness to participate	YY1	−1.043	1.707
	YY2	−0.749	1.221
	YY3	−0.941	1.853
	YY4	−0.802	0.851
Participatory behavior	XW1	−0.888	0.766
	XW2	−0.648	−0.151
	XW3	−0.851	0.454

### 4.2. Model fitness evaluation

**Fig 2:**
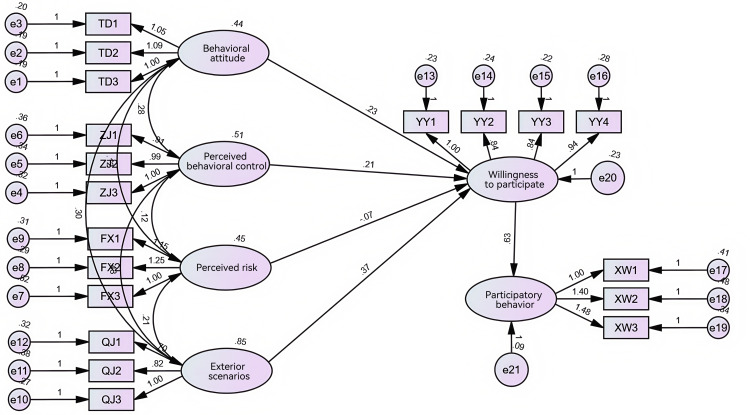
Structural equation modeling diagram.

As shown in Fig 2, this study used AMOS 24.0 software to construct a structural equation model and analyze consumer willingness and behavior to participate in virtual CSR activities. From the model fit test results in Table 10, it can be seen that CMIN/DF (chi-square to degrees of freedom ratio) = 2.024, which is within the range of 1–3, and RMSEA (root mean square error of approximation) = 0.067, which is less than 0.08. The test results of IFI, TLI, and CFI all reached above 0.9. Therefore, the SEM model has a good fit.

**Table 10:** Results of model fitness evaluation.

Index	Reference standards	Measured results
CMIN/DF	1-3 is excellent and 3–5 is good	2.024
RMSEA	< 0.05 is excellent and < 0.08 is good	0.067
IFI	> 0.9 is excellent and > 0.8 is good	0.940
TLT	> 0.9 is excellent and > 0.8 is good	0.926
CFI	> 0.9 is excellent and > 0.8 is good	0.939

### 4.3. Direct effect test

In this study, the following hypotheses were accepted: H1, H2, H3, and H5, when the significance level p-value was < 0.05 and the standardized path coefficient β > 0; and H4, when the p-value was < 0.05 and the standardized path coefficient β < 0. According to the results in Table 11, the standardized path coefficients of behavioral attitudes, perceived behavioral control, and external scenarios are 0.214, 0.205, and 0.473 respectively, all of which are greater than 0, and the p-value < 0.05. Therefore, behavioral attitudes, perceived behavioral control, and external scenarios significantly and positively influence consumer willingness to participate. H1, H2, and H3 are all valid. The standardized path coefficient of perceived risk is −0.061, which is less than 0, but the p-value > 0.05. Therefore, perceived risk does not significantly and negatively influence consumer willingness to participate, and H4 is not valid. The standardized path coefficient of willingness to participate is 0.828, which is greater than 0, and the p-value < 0.05. Therefore, consumer willingness to participate significantly and positively influences participatory behavior, and H5 is valid. In summary, H1, H2, H3, and H5 are valid, while H4 is not valid.

**Table 11:** Results of direct effect test.

Path relationship			Normalized path coefficients	S.E.	C.R.	p
Willingness to participate	←	Behavioral attitudes	0.214	0.082	2.822	0.005
Willingness to participate	←	Perceived behavioral control	0.205	0.098	2.095	0.036
Willingness to participate	←	Perceived risk	−0.061	0.066	−0.989	0.323
Willingness to participate	←	Exterior scenarios	0.473	0.071	5.145	***
Participatory behavior	←	Willingness to participate	0.828	0.071	8.858	***

### 4.4. Mediating effect test

The results of the direct effect test indicate that H4 is not supported, suggesting that perceived risk does not significantly have a negative impact on consumer willingness to participate in virtual CSR activities. Consequently, the willingness to participate does not serve as a mediator between perceived risk and participatory behavior, leading to the rejection of H9. Behavioral attitudes, perceived behavioral control, and external scenarios significantly influence the willingness to participate. Moreover, the willingness to participate significantly affects participatory behavior. Hence, it is necessary to analyze the mediating effect of the willingness to participate along these three paths.

The model employed in this study encompasses three paths. Each path was rigorously tested one by one using the Bootstrap method with a 95% confidence level and 2000 repeated samplings. The results are presented in Table 12. For the three paths related to behavioral attitudes, perceived behavioral control, and external scenarios, the indirect effects do not include zero within the 95% confidence interval. This finding clearly demonstrates that the willingness to participate exerts a significant mediating effect between behavioral attitudes, perceived behavioral control, external scenarios, and participatory behavior. In other words, H6, H7, and H8 are supported. However, since the direct effects within the paths of behavioral attitudes and external scenarios include zero within their respective 95% confidence intervals, it can be concluded that consumer willingness to participate in virtual CSR activities plays a full mediating role between behavioral attitudes, external scenarios, and participatory behavior. In contrast, the willingness to participate only plays a partial mediating role between perceived behavioral control and participatory behavior.

**Table 12:** Results of Bootstarp mediating effect test.

Mediation paths	Parameter	Estimate	Lower	Upper	p	Proportion of relative mediating effect
TD-YY-XW	Indirect effects	0.357	0.202	0.561	0.001	86%
	Direct effects	0.057	−0.085	0.287	0.392	
	Total effect	0.415	0.206	0.662	0.001	
ZJ-YY-XW	Indirect effects	0.307	0.204	0.47	0.000	61%
	Direct effects	0.196	0.067	0.407	0.003	
	Total effect	0.502	0.332	0.689	0.001	
QJ-YY-XW	Indirect effects	0.283	0.182	0.442	0.000	79%
	Direct effects	0.072	−0.069	0.251	0.271	
	Total effect	0.356	0.24	0.498	0.001	

## 5. Discussion and conclusions

This study, on the basis of the planned behavior theory model, introduces the variable of perceived risk and constructs a theoretical research model of consumer willingness and behavior to participate in virtual CSR activities. Then, it designs measurement scales and questionnaires, collects questionnaire data through Internet social platforms, and uses the structural equation model to test the theoretical model and various hypotheses.

The results of the hypothesis testing indicate that H1, H2, H4, and H5 are accepted. This suggests that behavioral attitudes, perceived behavioral control, and external scenarios all have a significant and positive effect on consumer willingness to participate in virtual CSR activities. The path coefficients in the constructed structural equations show that the standardized path coefficients of behavioral attitudes, perceived behavioral control, and external scenarios on consumer willingness to participate in virtual CSR activities are 0.214, 0.205, and 0.473 respectively. Additionally, the standardized path coefficient of consumer willingness to participate in virtual CSR activities on participatory behavior is 0.828. This indicates that willingness to participate significantly and positively influences consumer engagement behavior.

As evidenced by the analysis results, the external situational variable has the highest standardized path coefficient, indicating that its influence on consumer willingness to participate in virtual CSR activities is more pronounced than that of other factors. The external scenario variable mainly refers to the impact of others’ choices and awareness on the subject. The findings suggest that consumers may be subject to herd mentality when participating in virtual CSR activities. External factors, such as recommendations from friends and family and media publicity, can influence consumers. They are likely to have a significant impact on consumer willingness to participate in virtual CSR activities.

The standardized path coefficients of behavioral attitude and perceived behavioral control on consumer willingness to participate are 0.214 and 0.205, respectively. Since the difference between the two coefficients is negligible, these two variables have similar effects on consumer willingness to participate. Behavioral attitude refers to consumers’ perception of virtual CSR activities. The results show that when consumers hold a positive attitude towards virtual CSR activities, they are more inclined to participate. The positive effect of perceived behavioral control on willingness to participate indicates that when consumers perceive greater self-control over their participation in virtual CSR activities, their willingness to participate is stronger.

The standardized path coefficient results of willingness to participate on participatory behavior show that although consumer willingness to participate in virtual CSR activities can significantly promote the generation of participatory behaviors, a small proportion of the willingness to participate will not be translated into actions. This is because willingness to participate is not equivalent to participatory behavior. There are still some influencing factors that impede the transformation from willingness to behavior.

The results show that the impact of perceived risk on consumer willingness to participate in virtual CSR activities is not statistically significant. This may be because when enterprises carry out virtual CSR activities, the display of the activity process and results meets consumers’ needs, and the activity ensures consumers’ information security. Consequently, consumers’ perception of risk is relatively low and does not significantly affect their willingness to participate. We suppose that within the specific context of low-involvement virtual CSR activities, the inherent level of perceived risk may be sufficiently low that its direct inhibitory effect on intention is attenuated. More importantly, the motivational override mechanism proposed that the salient positive forces of attitude, subjective norm, and perceived behavioral control in prosocial domain may actively neutralize or outweigh general risk concerns during the intention-formation stage, suggesting a motivation-dominant decision paradigm [36]. This finding prompts a reconceptualization of the theoretical role of perceived risk in this context. Rather than acting as a direct antecedent to intention, it may function as a moderator of the intention-behavior relationship or as an influencer of more proximal factors such as trust.

The mediation effect test shows that consumer willingness to participate in virtual CSR activities acts as a complete mediator between behavioral attitudes, external scenarios and participatory behaviors. This means that behavioral attitudes and external scenarios affect participatory behavior indirectly by influencing their willingness to participate in virtual CSR activities. In contrast, consumer willingness to participate plays a partial mediating role between perceived behavioral control and participatory behavior. That is, perceived behavioral control will directly affect participatory behavior and also indirectly affect them through consumer willingness to participate.

## 6. Implications

The findings of this study offer insights for enterprise management. Firstly, it is imperative that companies innovate the forms of their virtual CSR activities. Our analysis confirms that perceived behavioral control significantly and positively influences the willingness to participate (H2, β = 0.205, p = 0.036). To maximize this effect, enterprises should focus on lowering the perceived barriers to entry. The term “perceived behavioral control” refers to consumers’ self-control over virtual CSR activities. In this study, perceived behavioral control mainly involves three factors: the degree of difficulty of virtual CSR activities, the time investment in participating in virtual CSR activities, and the degree of difficulty in collecting information related to virtual CSR activities. Streamline multi-step tasks into one-click actions or default settings. This directly minimizes the perceived effort, which is also related to the risk dimension of “requiring too much time and effort” (FX2). Embed brief tutorial videos (under 15 seconds) or dynamic guides at key interaction points. This proactively provides the information needed to participate, enhancing consumers’ confidence and control. Consequently, when designing virtual CSR activities, enterprises should reduce the complexity of each link in the virtual CSR activities and simplify the game or other steps in the process, enabling consumers to easily understand how to participate. Concurrently, enterprises should provide more explanations about virtual CSR activities or related content during the activity to help consumers understand better.

Secondly, upgrade promotional strategies to leverage attitude and, more importantly, social influence. While Behavioral Attitude positively affects participation intention (H1, β = 0.214, p = 0.005), our results identify external scenarios (social influence) as the strongest predictor (H3, β = 0.473, p < 0.001). Therefore, promotional efforts must evolve from merely stating benefits to actively shaping social norms. This could involve placing promotional content about virtual CSR activities in mobile applications with a large user base, displaying relevant promotion on the homepage of the enterprise’s official channels, messaging should connect individual action to collective identity and collaborating with other popular brands to increase the popularity of virtual CSR activities.

Thirdly, enterprises should make full use of consumer social network relationships. External circumstances (i.e., social influence) constitute the most potent factor driving willingness (H3, β = 0.473). Implementation strategies should transcend generic encouragement by designing quantifiable, visualisable social proof mechanisms. Consumers show a herd mentality when participating in virtual CSR activities, especially when those around them are also involved. This can increase consumer willingness to participate. It is therefore crucial to value the evaluations of virtual CSR activities by participants. Enterprises should design relevant mechanisms to encourage participants to share and recommend virtual CSR activities to their social circles via social media, and set up dedicated sections to publicize participants’ evaluations, so as to attract more new customers to participate.

## 7. Limitations and future research

This study is subject to several limitations. First, the research sample primarily consists of young, college-educated respondents, which may restrict the generalizability of the findings to broader age groups or different cultural settings. We strongly encourage future research to examine the robustness of our integrated model using more diverse demographic samples. Such efforts would help clarify which relationships hold universally and which may be specific to particular population segments. Second, the statistical results for Hypothesis H4 were non-significant. Although we have provided a theoretical discussion regarding this outcome, the underlying mechanism warrants further empirical validation. Subsequent studies should seek to refine the understanding of how perceived risk influences participation willingness, thereby contributing to a more comprehensive theoretical framework.

## Supporting information

S1 Data Data_PONE-D-25-34017. (XLSX)
